# Transgenic Testing Does Not Support a Role for Additional Candidate Genes in *Wolbachia* Male Killing or Cytoplasmic Incompatibility

**DOI:** 10.1128/mSystems.00658-19

**Published:** 2020-01-14

**Authors:** Jessamyn I. Perlmutter, Jane E. Meyers, Seth R. Bordenstein

**Affiliations:** a Department of Biological Sciences, Vanderbilt University, Nashville, Tennessee, USA; b Vanderbilt Microbiome Initiative, Vanderbilt University, Nashville, Tennessee, USA; c Department of Pathology, Microbiology, and Immunology, Vanderbilt University, Nashville, Tennessee, USA; d Vanderbilt Institute for Infection, Immunology, and Inflammation, Vanderbilt University, Nashville, Tennessee, USA; Princeton University

**Keywords:** *Wolbachia*, *Drosophila*, reproductive parasitism, male killing, cytoplasmic incompatibility, prophage WO, transgenics

## Abstract

Endosymbiotic bacteria in the genus *Wolbachia* remarkably infect nearly half of all arthropod species. They spread in part because of manipulations of host sexual reproduction that enhance the maternal transmission of the bacteria, including male killing (death of infected males) and unidirectional cytoplasmic incompatibility (CI; death of offspring from infected fathers and uninfected mothers). Recent discoveries identified several genes in prophage WO of *Wolbachia* (*wmk*, *cifA*, and *cifB*) that fully or partially recapitulate male killing or CI when transgenically expressed in Drosophila melanogaster. However, it is not yet fully resolved if other gene candidates contribute to these phenotypes. Here, we transgenically tested 10 additional gene candidates for their involvement in male killing and/or CI. The results show that despite sequence and protein architecture similarities or comparative associations with reproductive parasitism, transgenic expression of the candidates does not recapitulate male killing or CI. Sequence analysis across Wmk and its closest relatives reveals amino acids that may be important to its function. In addition, evidence is presented to propose new hypotheses regarding the relationship between *wmk* transcript length and its ability to kill a given host, as well as copy number of *wmk* homologs within a bacterial strain, which may be predictive of host resistance. Together, these analyses continue to build the evidence for identification of *wmk*, *cifA*, and *cifB* as the major genes that have thus far been shown to cause reproductive parasitism in *Wolbachia*, and the transgenic resources provide a basis for further functional study of phage WO genes.

**IMPORTANCE** *Wolbachia* are widespread bacterial endosymbionts that manipulate the reproduction of diverse arthropods to spread through a population and can substantially shape host evolution. Recently, reports identified three prophage WO genes (*wmk*, *cifA*, and *cifB*) that transgenically recapitulate many aspects of reproductive manipulation in Drosophila melanogaster. Here, we transgenically tested 10 additional gene candidates for CI and/or male killing in flies. The results yield no evidence for the involvement of these gene candidates in reproductive parasitism, bolstering the evidence for identification of the *cif* and *wmk* genes as the major factors involved in their phenotypes. In addition, evidence supports new hypotheses for prediction of male-killing phenotypes or lack thereof based on *wmk* transcript length and copy number. These experiments inform efforts to understand the full basis of reproductive parasitism for basic and applied purposes and lay the foundation for future work on the function of an interesting group of *Wolbachia* and phage WO genes.

## Abstract (precis)

*Wolbachia* are widespread bacterial endosymbionts that manipulate the reproduction of diverse arthropods to spread through a population and can substantially shape host evolution. Recently, reports identified three prophage WO genes (*wmk*, *cifA*, and *cifB*) that transgenically recapitulate many aspects of reproductive manipulation in Drosophila melanogaster. Here, we transgenically tested 10 additional gene candidates for CI and/or male killing in flies. The results yield no evidence for the involvement of these gene candidates in reproductive parasitism, bolstering the evidence for identification of the *cif* and *wmk* genes as the major factors involved in their phenotypes. In addition, evidence supports new hypotheses for prediction of male-killing phenotypes or lack thereof based on *wmk* transcript length and copy number. These experiments inform efforts to understand the full basis of reproductive parasitism for basic and applied purposes and lay the foundation for future work on the function of an interesting group of *Wolbachia* and phage WO genes.

## INTRODUCTION

Some of the most widespread microbial symbioses on the planet occur between invertebrates and various microbes that manipulate host reproduction (1). These reproductive parasites hijack host cellular processes and alter host reproduction to facilitate their spread. They include a variety of maternally inherited bacterial, fungal, and viral endosymbionts that infect a large number of arthropod hosts, including all major groups of insects and arachnids (2). Among these microbes, the most common are of the genus *Wolbachia*, which are obligate intracellular bacteria that manipulate host reproduction in a variety of ways (3, 4). There are at least four main phenotypes, including (i) male killing (selective killing of male hosts), (ii) feminization (physical development and reproduction of genetic males as females), (iii) parthenogenesis (asexual reproduction of females), and (iv) unidirectional cytoplasmic incompatibility (CI; death of offspring when infected males mate with uninfected females or infected females harboring incompatible strains and “rescue” from death in matings between parents infected with compatible strains). Each phenotype facilitates spread of the bacteria by either increasing the fitness of infected females through induction of a female-biased sex ratio (i to iii) or decreasing the fitness of uninfected females through a reduction in the abundance of viable offspring (iv).

Of these phenotypes, two in particular have current or potential use in arthropod pest and vector control efforts and are important to the basic biology of both host and microbe, making them the subject of diverse research interest. CI, the most widespread phenotype, is currently deployed in Aedes albopictus and A. aegypti mosquitoes to reduce the incidence of vector-borne diseases (5–8). These efforts have achieved early success (5, 8, 9), but there are potential challenges for widespread applications of CI-based vector control, including potential difficulty in spreading CI-*Wolbachia* strains to recalcitrant host species (10–12). In addition, male killing is a potential adjunctive or standalone control method. Although it has not yet been tested empirically in arthropods, population modeling suggests that male killing could be especially useful in a two-pronged approach alongside CI or sterile insect technique (SIT) (13). CI and male killing also have important consequences for host evolution and ecology. As CI can kill offspring from crosses between infected males and uninfected females or between infected males and females harboring incompatible strains of *Wolbachia*, CI can be a barrier to gene flow between populations or incipient species (14, 15). Male killing, on the other hand, may lead to evolutionary outcomes such as host extinction, loss of the male-killer (16), and host development of heritable resistance to male killing (11, 17, 18). In addition, female-biased populations may exhibit altered sexual selection. For example, infected Hypolimnas bolina female butterflies become more promiscuous, can form lekking swarms, and display mate-attracting behaviors (19, 20).

Given the aforementioned relevance to both applied and basic research, there is considerable interest in the genetics of reproductive parasitism (21) and in phage WO in particular (22–29). Phage WO has a unique genome among phages because it includes a eukaryotic association module (EAM) that is enriched with genes annotated or demonstrated to have eukaryotic function or homology (22). Many of the EAM genes are unique to this bacteriophage and putatively encode functions that underlie host-symbiont interactions. Indeed, the genes underlying the CI phenotype (*cifA* and *cifB* [*cytoplasmic incompatibility factors A* and *B*], loci WD0631 and WD0632) are just a few genes away from the male-killing gene candidate (*wmk* [*WO-mediated killing*], locus WD0626) in the EAM region of prophage WO (24–26). The two *cif* genes synthetically recapitulate the full CI and rescue phenotypes when expressed transgenically in Drosophila melanogaster (24, 26). Similarly, transgenic *wmk* expression specifically and consistently kills a third of male hosts and preferentially induces cytological defects in male embryos that are typical of natural infection (25). In addition, a *Spiroplasma* male-killing gene (encoding protein *Spiroplasma* androcidin [*Sp*AID]) was recently reported on a plasmid and likely functions via interference with host dosage compensation (21). Significantly, all of these genes are unique in nature, with specialized functions and no known homologs in other organisms, and thus represent new frontiers in understanding host-endosymbiont biology.

Importantly, although the genes thus far have been found to recapitulate several cytological, biochemical, and embryonic phenotypes of natural infection, the genetic basis of reproductive parasitism may not be fully resolved. Additional gene candidates from comparative genomic analyses exist, and modifier genes in phage WO or *Wolbachia* may alter the penetrance of the phenotypes. For example, the *wmk* gene is a candidate for *Wolbachia*-induced male killing due to its recapitulation of many aspects of the natural phenotype, including male-biased embryonic defects and lethality and associations between dosage compensation activity and DNA damage. However, *wmk* expression killed over a third of gene-expressing males instead of all males under the conditions tested thus far (25). The incomplete penetrance could be due to inadequate transgenic expression levels or patterns, host resistance, or involvement of another gene in the phenotype (25). Previous work tested different *wmk* expression levels, but results showed that increased expression levels led to a similar phenotype (25). Notably, there are many connections between the CI and male-killing cytological defects (such as chromatin bridging) (24, 25, 30, 31) that suggest they may have overlapping functions, but the basis of these connections remains unclear. In addition, many strains of *Wolbachia* are multipotent and thus induce either male killing or CI depending on the host or environment (11, 12, 18, 32). Further, a previous comparative genomic analysis of CI-associated genes demonstrated that *wmk* was shared across CI-causing genomes (24), and *cifA* was identified as a top candidate in a comparative genomic analysis of the genes underpinning male killing (25). Not all additional candidates have been tested and not all putative phenotypes have been investigated; thus, there may be other genes that recapitulate CI or male killing. The nature of any putative relationship between CI and male-killing genes is also unclear.

Several genes were previously identified that are moderately associated with reproductive parasitism in *Wolbachia*, but they were not empirically tested for function. These candidates were identified through similarity to genes encoding *Sp*AID or the CI proteins (21) or homology to the *wmk* gene or in previous comparative genomic analyses of genes associated with male killing (25). Here, we analyzed and transgenically tested these gene candidates for recapitulation of reproductive parasitism to assess the hypothesis that phage WO contains additional genes that mediate parasitism of host reproduction.

## RESULTS

### Many additional *w*Mel genes are candidates for reproductive parasitism.

**FIG 1:**
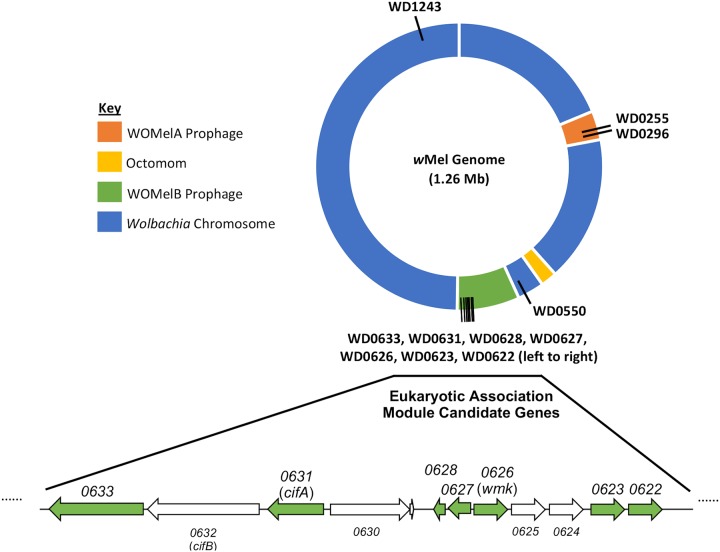
Map of gene candidates assessed for reproductive parasitism across the *w*Mel genome. Prophage WO regions are shown in their indicated colors. Gene positions, indicated by black lines, are approximate. The white line at the top indicates the first nucleotide position in the genome. The WOMelB prophage region is expanded below to show the relative positions of these genes. Genes are roughly to scale, with candidates shown in green and noncandidate genes in white. Different arrow directions indicate locations on opposite DNA strands.

Although several recent studies have identified genes that recapitulate reproductive parasitism phenotypes, additional male-killing candidates have been previously reported or are reported here, albeit some with lower support for a genotype-phenotype association (Table 1). The characteristics accounting for inclusion of these genes can be broken down into several categories: (i) predicted protein similarity to the *Sp*AID male-killing toxin, probability of type IV secretion, and presence of an operational taxonomic unit (OTU) deubiquitinase domain similar to the CI genes (WD0633); (ii) additional homologs of *wmk* within the *w*Mel *Wolbachia* genome (WD0622, WD0623, WD0255); (iii) candidates identified through a previous male-killing comparative genomic analysis (WD1243, WD0296, WD0550, WD0631 [*cifA*], WD0628, WD0627); and (iv) *wmk* with a putative alternative start codon (identified and described here). Of the loci identified, two are in the WOMelA prophage region, seven are in the WOMelB prophage region that includes the EAM, and two are in the *Wolbachia* chromosome of the *w*Mel strain but are in the prophage region of the *w*Bif male-killing *Wolbachia* strain of Drosophila bifasciata (Fig. 1).

**TABLE 1:** List of gene candidates for *Wolbachia*/phage WO male killing, their putative functions or domains, the basis for their inclusion, and any publications or figures in which they were identified as candidates^*a*^

Gene	Putative function(s) or domain(s)	Reason(s) for inclusion	Identifying publication or figure(s)
WD0626 (*wmk*)	HTH DNA-binding TF	Previous transgenic testing	25
WD0633	OTU, ankyrin repeats	Domains similar to *Sp*AID	21
		Previous correlation with CI	37
		Similar to known T4SS effectors	Fig. S2
WD0622	HTH DNA-binding TF	Homolog of *wmk*	25
WD0623	HTH DNA-binding TF	Homolog of *wmk*	25
WD0255	HTH DNA-binding TF	Homolog of *wmk*	25
WD1243	Putative phospholipase D or nuclease	Genomic analysis for MK candidates	25
WD0296	Recombination-promoting nuclease (Rpn)	Genomic analysis for MK candidates	25
WD0550	Ankyrin repeats	Genomic analysis for MK candidates	25
WD0628	Hypothetical protein	Genomic analysis for MK candidates	25
WD0627	Recombination-promoting nuclease (Rpn)	Genomic analysis for MK candidates	25
WD0626 (*wmk*), alternative start codon	HTH DNA-binding TF	Previous transgenic testing	Fig. 6, Fig. S3
WD0631 (*cifA*)	DUF, catalase-rel, STE TF	Similar phylogeny	Fig. 5

a catalase-rel, catalase-related; CI, cytoplasmic incompatibility; DUF, domain of unknown function; MK, male killing; STE, sterile-class transcription factor; TF, transcription factor; T4SS, type IV secretion system; HTH, helix-turn-helix; OTU, ovarian tumor.

### WD0633 in prophage WO does not transgenically recapitulate male killing or CI.

**FIG 2:**
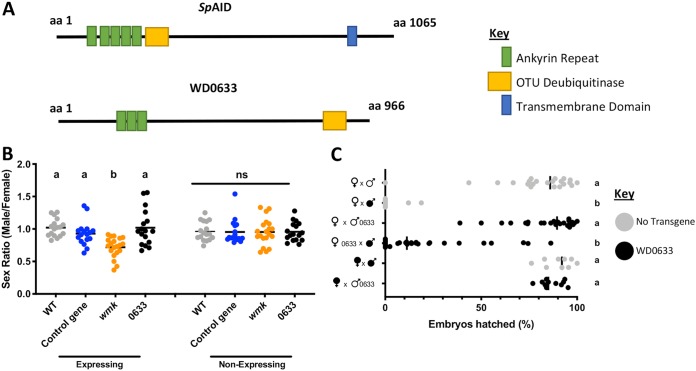
Transgenic expression of WD0633 does not recapitulate male killing or CI. (A) Diagrams of protein architecture using domains indicated from SMART (59) and HHpred (60) databases. (B) Sex ratios of adult flies either expressing (*Act5c*-Gal4) or not expressing (CyO) the indicated genes. Each sample point represents the adult offspring produced by a replicate family of 10 mothers and 2 fathers, with expressing and nonexpressing flies of a given genotype being siblings. Bars represent the mean sex ratios. Statistics are based on a Kruskal-Wallis one-way ANOVA followed by Dunn’s correction across either expressing or nonexpressing flies. ns, results were not statistically significant. (C) Hatch rate of embryos with infected (filled sex symbol) or uninfected (unfilled sex symbol) flies expressing or not expressing an indicated gene with the *nanos*-Gal4:VP16 gonad-specific driver. Bars represent the median hatch rate. Each dot represents the hatch rate of offspring of a single male and female. Black dots indicate a cross with WD0633, and gray dots indicate crosses without transgenes. Statistics are based on a Kruskal-Wallis one-way ANOVA followed by Dunn’s correction.

Candidate gene WD0633 was identified in a previous publication that reported *Sp*AID is the *Spiroplasma* male-killing toxin (21). Although the authors did not find any homologs in *Wolbachia* based on the full gene sequence, they noted that WD0633 shares putative protein domain features such as an OTU deubiquitinase domain and several ankyrin repeats. Despite this similarity in putative domain identities, the overall protein architecture is different due to the presence of fewer putative ankyrin repeats and different localization of the deubiquitinase domain in WD0633 (Fig. 2A). In addition, results of *Sp*AID BLASTP analysis do not show any full homologs to the gene in organisms other than *Spiroplasma*. However, genes on mobile elements such as phages or plasmids (*Sp*AID is reportedly on a plasmid) are often developed by fusion of gene sequences from several different sources (22). Therefore, we performed BLASTP searches on different regions of the protein. Results showed that the OTU domain had weak homology to *Wolbachia* proteins, while other regions had homology only to *Spiroplasma* proteins. An unrooted Bayesian tree demonstrates that homologs cluster by bacterial genus (see Fig. S1A in the supplemental material). The homology suggests that there may have been gene exchanges between these two genera, although the direction of any putative gene exchange was not determined. The notion of the likelihood of a gene transfer event is not unreasonable given that the two bacteria can infect the same host organisms (33, 34). In contrast, the WD0633 full protein and the OTU domain have no significant homology to *Spiroplasma* proteins, leaving no indication of a relationship with *Spiroplasma*. Thus, there may be a link between *Sp*AID and other *Wolbachia* protein sequences, but the results support previous findings indicating that WD0633 and *Sp*AID are not true homologs (21) and that WD0633 is not a homolog of any other known *Spiroplasma* protein.

10.1128/mSystems.00658-19.1 FIG S1 *Sp*AID is similar in sequence and structure to *Wolbachia* proteins. (A) A Bayesian phylogeny of the OTU domain of *Sp*AID and homologous sequences from NCBI based on a 54-aa alignment using the cpRev model of evolution. Posterior probability values are shown on the branches. Purple branches correspond to *Wolbachia* sequences, and green branches correspond to *Spiroplasma* branches. (B) Protein structure predictions from the Phyre2 web portal (35). Images are of the most likely three-dimensional (3D) structures of *Sp*AID and WD0633 determined by Phyre2. *Sp*AID has 100% confidence, 54% coverage, and 9% sequence identity (id) to the top model based on the c4cj9A template (BurrH DNA-binding protein from Burkholderia rhizoxinica in apo form). WD0633 has 100% confidence, 58% coverage, and 10% id to the same model. Colors are in order of the rainbow from the 5′ end to 3′ end of the modeled portion of the genes. Download FIG S1, TIF file, 0.9 MB. Copyright © 2020 Perlmutter et al. 2020 Perlmutter et al. This content is distributed under the terms of the Creative Commons Attribution 4.0 International license.

Interestingly, despite no evidence of a shared ancestry and despite the significant differences in protein sequences (1.7% pairwise amino acid identity), the two proteins have similar protein structure predictions (Fig. S1B). Phyre2 protein modeling (35) indicates that both have putative similarity to the BurrH DNA-binding protein from Burkholderia rhizoxinica, a symbiont of *Rhizopus* microspores (36), which is intriguing as Wmk has two predicted helix-turn-helix (HTH) DNA-binding domains (25). In both cases, BurrH was predicted to be the best model template, resulting in nearly identical structural predictions for the modeled regions of the two proteins (Fig. S1B). This suggests that they may share similar functions due to architectural similarity despite disparate sequences. This is additionally intriguing, given that *Sp*AID functions as a male-killing toxin but WD0633 is not present in all male-killing strains (it is absent from *w*Rec of Drosophila recens) and thus would not be predicted to have a male-killing function in *Wolbachia*.

Beyond male killing, WD0633 was also identified as a candidate for CI in an earlier comparative genomic analysis, but it failed to recapitulate the phenotype upon transgenic testing in D. melanogaster (37). However, this was done with a transgene driver that has ubiquitous expression in all tissues rather than specifically in the gonads. In addition, an OTU deubiquitinase domain was previously reported in the CI-causing *cifB* gene (27) based on *in vitro* and yeast studies, though similar effects were not confirmed *in vivo* in flies. Further, WD0633 has multiple motifs and domains that are enriched in type IV secretion system (T4SS) effectors such as eukaryotic-like domains (present in the EAM), three EPIYA domains, a coiled-coil, C-terminal basicity, and global hydrophobicity (Fig. S2). On the basis of these features, Searching Algorithm for Type IV Effector Proteins 2.0 (S4TE) calculates a high probability of type IV secretion (see Table S1 in the supplemental material) (38, 39). All of these factors make WD0633 likely to function in the eukaryotic host and thus a particularly interesting candidate for study in reproductive parasitism. On the basis of the reasons cited above, we tested the transgene for recapitulation of male killing with a ubiquitous transgene driver and CI with a gonad-specific driver.

10.1128/mSystems.00658-19.2 FIG S2 Type IV secretion system (T4SS) motifs in WD0626. Domains and motifs identified by the S4TE program are labeled (39). C-terminal basicity refers to the entire C-terminal region, and “Global Hydrophobicity” refers to the entire protein. The black line indicates the protein sequence, and the other lines and boxes indicate T4SS effector-like features. Download FIG S2, TIF file, 0.1 MB. Copyright © 2020 Perlmutter et al. 2020 Perlmutter et al. This content is distributed under the terms of the Creative Commons Attribution 4.0 International license.

10.1128/mSystems.00658-19.4 TABLE S1 List of *w*Mel genes that scored above the threshold value in the S4TE algorithm for type IV secretion system effectors by locus tag and score. The entire *w*Mel genome was searched and yielded 148 putative effectors among 1,195 total proteins and a threshold score of 72. Download Table S1, DOCX file, 0.02 MB. Copyright © 2020 Perlmutter et al. 2020 Perlmutter et al. This content is distributed under the terms of the Creative Commons Attribution 4.0 International license.

To test for male-killing function, we transgenically expressed WD0633 in D. melanogaster flies using a ubiquitous driver (*Act5c*-Gal4/CyO) under the same conditions as those previously used to evaluate *wmk* (25). Transgenic WD0633 expression did not cause a biased sex ratio in adult offspring, similarly to a control transgene and wild-type (WT) flies, indicating no recapitulation of male killing in this system. This contrasts with transgenic *wmk* expression, which displayed the expected biased sex ratio, whereas nonexpressing siblings did not display a biased sex ratio (Fig. 2B). To test for putative CI function, we expressed WD0633 using the gonad-specific driver *nanos*-Gal4:VP16 in ovaries and testes of adults. We then crossed adults of the indicated genotypes together (infected, uninfected, or uninfected expressing WD0633 in gonads) and determined the proportion of embryos hatching into larvae as a measure of CI. However, expression in male gonads did not recapitulate CI, and expression in female gonads did not recapitulate rescue (Fig. 2C).

### Divergent *wmk* homologs in *w*Mel do not transgenically recapitulate male killing.

**FIG 3:**
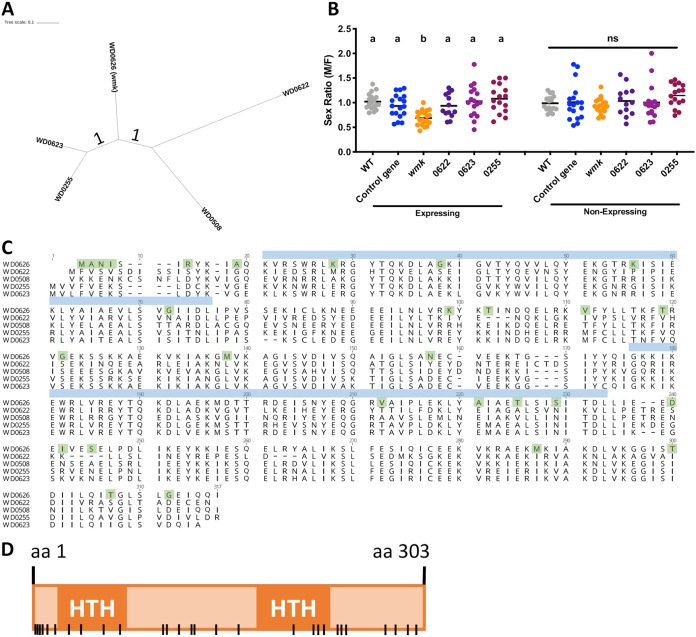
Transgenic expression of *w*Mel *wmk* homologs does not recapitulate male killing. (A) Nucleotide phylogeny of *w*Mel *wmk* homologs. (B) Sex ratios of adult flies either expressing (*Act5c*-Gal4) or not expressing (CyO) the indicated genes. Each sample point represents the adult offspring produced by a replicate family of 10 mothers and 2 fathers, with expressing and nonexpressing flies of a given genotype being siblings. Bars represent the mean sex ratios. Statistics are based on a Kruskal-Wallis one-way ANOVA followed by Dunn’s correction across either expressing or nonexpressing flies. (C) Amino acid alignment of Wmk (WD0626) and its homologs in *w*Mel. Green highlights indicate amino acids unique to Wmk. Blue boxes indicate the NCBI-predicted position of the HTH DNA-binding domains (above the indicated amino acids). (D) Schematic of amino acids unique to WD0626 (Wmk) across the protein sequence, as indicated by black lines. Locations are approximate.

The *w*Mel *Wolbachia* genome *of* D. melanogaster contains the eukaryotic association module (EAM) region in prophage WO, which contains genes with putative or demonstrated eukaryotic functions or homology (22). The previously identified *wmk* gene resides in this region, as do several of its homologs. Indeed, some *Wolbachia* genomes contain multiple copies of *wmk* homologs that have apparently arisen by duplication and divergence or by integration of multiple phages. For example, *w*Mel contains four additional homologs, *w*Inn of D. innubila contains three, *w*Bor *of* D. borealis contains three, and *w*Bol1b of Hypolimnas bolina butterflies contains seven (25). *w*Mel contains four of these homologs, which share 65% to 81% pairwise nucleotide sequence identity with *wmk* and encode similar proteins that are all predicted to contain the two HTH DNA-binding domains annotated in *wmk* (Fig. 3A) (25). Although *wmk* is the only *w*Mel homolog shared across all male-killer genomes, we assessed the others in the *w*Mel genome for putative male-killing function in this host as these copies may share the ability to kill males. One of the homologs, WD0508, was previously tested and did not recapitulate the phenotype (25). Here, we tested an additional three homologs: WD0622, WD0623, and WD0255 (Fig. 3B). All are prophage WO genes, and the first two are in the same prophage WOMelB EAM region as the *wmk* gene, while WD0508 (octomom region) and WD0255 (WOMelA) are in other regions (Fig. 1). However, upon transgenic expression using the *Act5c*-Gal4/CyO driver described above, the newly tested transgenes did not recapitulate a sex-ratio bias, indicating an inability to cause male killing in this system (Fig. 3B). On the basis of this functional analysis, we performed an alignment of the amino acid sequences of Wmk and its homologs and identified 28 amino acid (aa) residues unique to Wmk (Fig. 3C) that may account for its specific ability to transgenically kill males. These amino acids are spread throughout the protein and do not yet identify a specific protein region crucial for male death (Fig. 3D).

### Additional male-killing gene candidates identified via comparative genomics do not recapitulate male killing.

**FIG 4:**
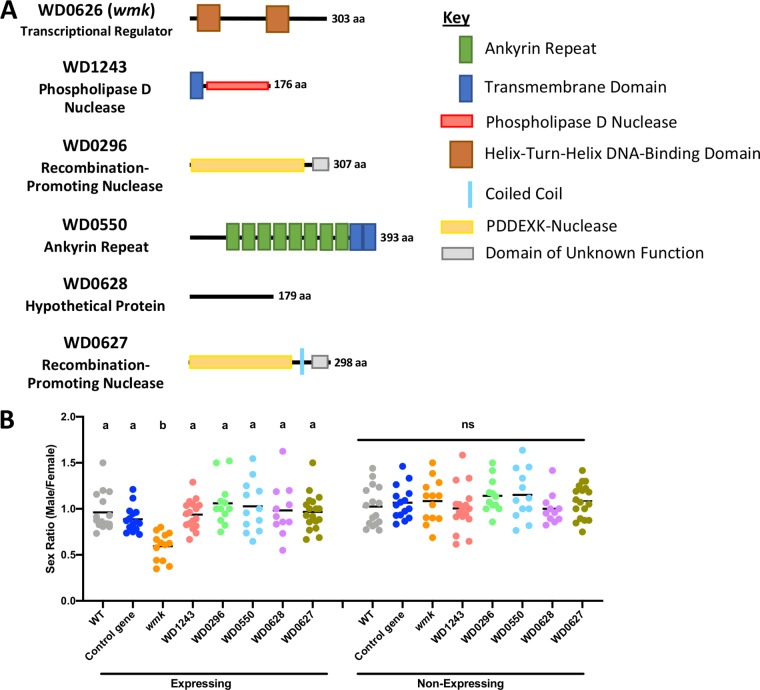
Additional male-killing gene candidates do not induce a biased sex ratio with transgenic expression. (A) Diagrams of protein architecture using the indicated domains from the SMART (59) and HHpred (60) databases. (B) Sex ratios of adult flies either expressing (*Act5c*-Gal4) or not expressing (CyO) the indicated genes. Each sample point represents the adult offspring produced by a replicate family of 10 mothers and 2 fathers, with expressing and nonexpressing flies of a given genotype being siblings. Bars represent the mean sex ratios. Statistics are based on a Kruskal-Wallis one-way ANOVA followed by Dunn’s correction across either expressing or nonexpressing flies.

In our previous study, we performed a comparative genomic analysis to identify genes associated with male-killer genomes (25). Among these were *wmk* and *cifA*, the latter of which functions in the induction and rescue of CI. An additional five candidates with a variety of putative functions were not previously tested, including WD1243 (putative endonuclease or phospholipase D domain, NCBI conserved domain E = 2.22 × 10^−73^), WD0296 (recombination-promoting PDDEXK family nuclease, NCBI conserved domain E = 4.50 × 10^−50^), WD0550 (ankyrin repeat, NCBI conserved domain E = 1.98 × 10^−34^), WD0628 (hypothetical protein), and WD0627 (recombination-promoting PDDEXK family nuclease, NCBI conserved domain E = 2.95 × 10^−55^) (Fig. 4A). Of these genes, one (WD0296) is in a prophage WO region different from that harboring *wmk* (WOMelA) and two more (WD1243 and WD0550) are in the *Wolbachia* chromosome of *w*Mel (Fig. 1). Of the two that are not in prophage WO regions in *w*Mel, both are phage genes in other strains, including the *w*Bif male-killing strain of D. bifasciata (25). One of the two, WD0550, contains ankyrin repeats that are abundant in phage WO genes and that have been implicated in reproductive parasitism (37, 40–42). The other, WD1243, is a putative endonuclease or phospholipase D gene that encodes a product with homology to proteins in *Rickettsia* and *Coxiella*, among others, according to a BLASTP search. These organisms are common parasites that contain plasmids and other mobile elements (43, 44), but ancestral *Ehrlichia* and *Anaplasma* species do not contain the gene. In addition, two candidate genes are present in the same EAM region between *wmk* and the *cifA* and *cifB* genes and therefore might have connections to parasitism due to proximity (WD0627, WD0628). However, when expressed transgenically with a ubiquitous driver, none of the five additional genes induced a biased sex ratio, indicating that they do not recapitulate male killing when expressed on their own (Fig. 4B).

### *wmk* and *cifA* do not transgenically function together in reproductive parasitism.

Our previous comparative genomic analysis identified *cifA* as a top candidate for male killing (25). In addition, a similar analysis of CI candidate genes also identified *wmk* as part of a “core” set of CI sequences shared across multiple CI-causing strains (24). Therefore, both genes appeared to be candidates in both reproductive parasitism analyses. Importantly, in the highly reduced prophage region in the *w*Rec male-killing and CI-inducing strain of D. recens, there are only 10 prophage WO genes remaining in the region that map to the WOMelB prophage of *w*Mel (23). In comparison, *w*Mel contains 88 genes in this region. Both *wmk* and *cifA* are included among the 10 genes remaining in *w*Rec; however, there are no additional *wmk* homologs. Indeed, *cifA* and *wmk* commonly co-occur in *Wolbachia* phage WO regions and are located near each other in some male-killer genomes (25). These co-occurrences in genomes indicate that the two may have similar origins or functions.

**FIG 5:**
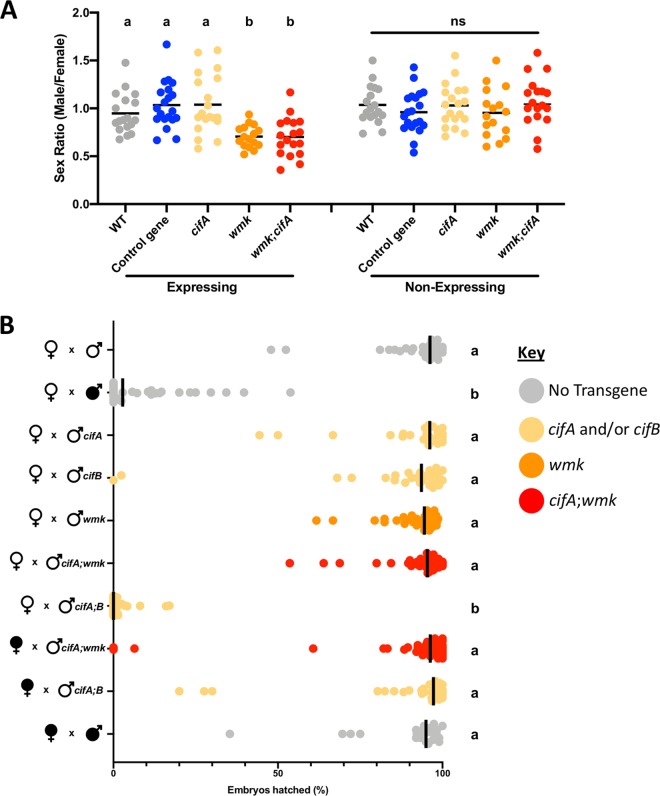
Coexpression of *cifA* and *wmk* neither enhances the *wmk* sex ratio bias nor recapitulates CI induction. (A) Sex ratios of adult flies either expressing (*Act5c*-Gal4) or not expressing (CyO) the indicated genes. Each sample point represents the adult offspring produced by a replicate family of 10 mothers and 2 fathers, with expressing and nonexpressing flies of a given genotype being siblings. Bars represent the mean sex ratios. Statistics are based on a Kruskal-Wallis one-way ANOVA followed by Dunn’s correction across either expressing or nonexpressing flies. (B) Hatch rate of embryos with infected (filled sex symbol) or uninfected (unfilled sex symbol) flies expressing or not expressing an indicated gene with the *nanos*-Gal4:VP16 gonad-specific driver. Bars represent the median hatch rate. Each dot represents the hatch rate of offspring of a single male and female. Colors indicate the presence or absence of the transgenes as indicated in the key. Statistics are based on a Kruskal-Wallis one-way ANOVA followed by Dunn’s correction.

Due to their coappearance in two analyses of CI and male killing, their presence in a reduced genome that causes both phenotypes, and their close proximity in several *Wolbachia* genomes, we assessed whether they function together transgenically by expressing single and dual *cifA* and *wmk* genes. To test for induction of male killing, we expressed the genes singly or together with the ubiquitous *Act5c*-Gal4/CyO driver and measured the sex ratios of the surviving adults (Fig. 5A). *cifA* does not induce a biased sex ratio on its own, and it neither enhances nor inhibits the ability of *wmk* to cause a biased sex ratio. Therefore, *cifA* is unlikely to play a role in male killing. To test for induction of CI, we performed a hatch rate experiment with the gonad-specific driver *nanos*-Gal4:VP16 to drive expression of each gene singly or together and measured the number of eggs that hatched into larvae to quantify CI induction (Fig. 5B). Despite CI induction resulting from either *w*Mel infection or coexpression of the CI-inducing *cifA*-*cifB* gene combination in males, neither individual expression nor coexpression of the *cifA* and *wmk* genes induced CI. This indicates that *wmk* is also likely not involved in induction of CI, as it did not reduce the rate of hatching to larvae even when expressed in adult gonads alone or with *cifA*. Only the two CI-inducing genes, *cifA* and *cifB*, were able to fully recapitulate CI induction when expressed together. Therefore, despite the many connections between *wmk* and *cifA*, the data do not support the hypothesis that they function together in reproductive parasitism.

### Transgenic expression with alternative transcripts of *wmk* results in loss of the sex ratio phenotype.

**FIG 6:**
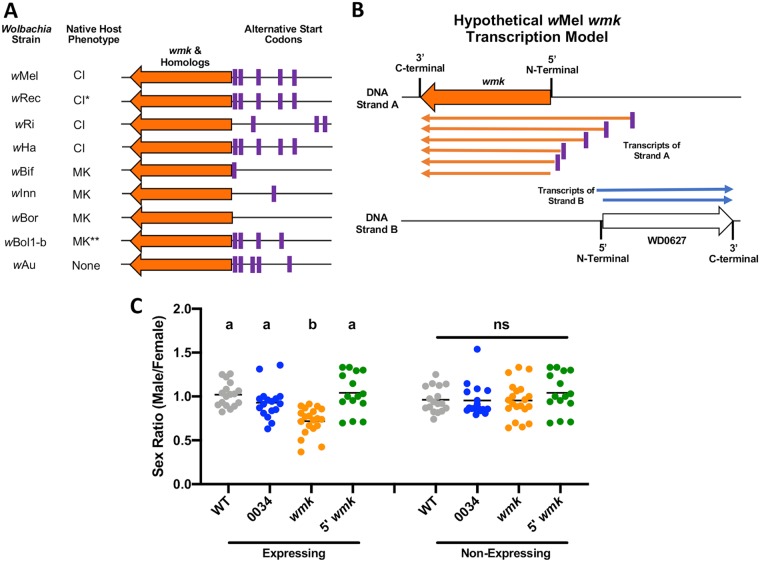
Expression of *wmk* with an alternative upstream start codon results in loss of a sex ratio bias. (A) Diagram of locations of alternative start codons up to 100 bp upstream of *wmk* or its homologs in the indicated strains. Purple stripes indicate the codons (not to scale). *, *w*Rec causes CI in its native host but can cause male killing when introgressed into a sister species. **, *w*Bol1-b natively infects a nondrosophilid host, the *Hypolimnas bolina* blue moon butterfly, while all other strains in the diagram infect drosophilid hosts. (B) Diagram of hypothetical model where multiple *wmk* transcripts of various lengths are expressed. (C) Sex ratios of adult flies either expressing (*Act5c*-Gal4) or not expressing (CyO) the indicated genes. Each sample point represents the adult offspring produced by a replicate family of 10 mothers and 2 fathers, with expressing and nonexpressing flies of a given genotype being siblings. Bars represent the mean sex ratios. Statistics are based on a Kruskal-Wallis one-way ANOVA followed by Dunn’s correction across either expressing or nonexpressing flies.

Previous testing of the *wmk* transgene was performed using its annotated methionine start codon in the NCBI database, resulting in a protein of 303 amino acids (aa) (25). However, additional inspection of the genome identified an alternative start codon (leucine) 9 aa upstream that could putatively produce a 312-aa protein, as bacteria may use noncanonical codons for proteins (45). To more broadly assess genomes for the presence of alternative start codons, we also analyzed the genomes of four CI-causing and four male-killing *Wolbachia* strains for the presence of any alternative start codons within 100 bp upstream of the annotated start codons (Fig. 6A; see also Table S2). Indeed, CI-causing strains had between 3 and 5 alternative start codons (mean, 4.5) in this region, while male-killers had between 0 and 4 (mean, 1.5). There is additional nuance to this pattern, as *w*Rec is natively a CI-causing strain, but can cause male killing in a nonnative host, and *w*Bol1-b is the only strain in this group that infects a nondrosophilid host. However, the presence of putative additional start codons is consistent with the hypothesis that there may be expression of alternative transcripts of *wmk* in certain strains or hosts, and it could relate to the presence, or lack thereof, of parasitism phenotypes in a given host. Notably, the WD0508 *wmk* homolog in *w*Mel is annotated with a noncanonical GTG start codon, as are many other *w*Mel genes, so the strain likely expresses at least one homolog of *wmk* with alternative codons. As one potential model, it is possible that expression of *wmk* results in several different transcripts of various lengths, which is known to occur with some bacterial genes (Fig. 6B). Indeed, inspection of publicly available *w*Mel transcription data shows that young embryos transcribe upstream of the annotated *wmk* start codon in many samples (Fig. S3A) (46).

10.1128/mSystems.00658-19.3 FIG S3 Alternative transcripts of *wmk* may have different structures and functions. (A) Image of gene transcription of *w*Mel-infected Drosophila melanogaster embryos from the Genome Browser database of the University of California, Santa Cruz (UCSC) (68). Each row represents a sample of the indicated embryo age. Red indicates transcription on the same strand as *wmk*, and blue indicates transcription on the opposite strand with WD0627. Most samples have transcription upstream of the annotated *wmk* start codon. (B) Sex ratios of adult flies either expressing (*Act5c*-Gal4) or not expressing (CyO) the indicated genes, including the 5′HA tag. (C) Sex ratios of adult flies either expressing (*Act5c*-Gal4) or not expressing (CyO) the indicated genes, including the 3′HA tag. Each sample point represents the adult offspring produced by a replicate family of 10 mothers and 2 fathers, with expressing and nonexpressing flies of a given genotype being siblings. Bars represent the mean sex ratios. Statistics are based on results from a Kruskal-Wallis one-way ANOVA followed by Dunn’s correction across either expressing or nonexpressing flies. (D) Predicted RNA secondary structures of *wmk* and *wmk* with the alternative start codon tested as described for Fig. 6C. The predicted structure was produced by the RNAfold web server (69) and represents the MFE secondary structure. The colors are indicative of base pair probabilities, with 0 probability represented in blue and high probability (1) represented in red. Download FIG S3, TIF file, 1.3 MB. Copyright © 2020 Perlmutter et al. 2020 Perlmutter et al. This content is distributed under the terms of the Creative Commons Attribution 4.0 International license.

10.1128/mSystems.00658-19.5 TABLE S2 List of alternative start codons in the same frame as the annotated codon in *w*Mel and male-killer genomes of *Drosophila* hosts. A star symbol (*) indicates the primary start codon annotated by Geneious. Total length data include the length of the stop codon. Download Table S2, DOCX file, 0.01 MB. Copyright © 2020 Perlmutter et al. 2020 Perlmutter et al. This content is distributed under the terms of the Creative Commons Attribution 4.0 International license.

To functionally test this model, we transgenically expressed an alternative version of Wmk with 9 additional amino acid residues, as this variant had an alternative start codon (TTG) in model prokaryotes that was more commonly used than others in the upstream region. Although *Wolbachia* start codon usage is unexplored, ATG (81.8%), GTG (13.8%), and TTG (4.3%) are the most common alternatives in model prokaryotes, and they are used in nearly all cases, with other codons occurring less than 1% of the time (45). Upon codon optimization and transgenic expression in D. melanogaster with a methionine start codon for optimal expression in this eukaryotic organism, the gene loses the ability to cause a biased sex ratio (5′ *wmk*; Fig. 6C). Though expression of the annotated 303-aa Wmk protein replicated the biased sex ratio phenotype as in previous experiments, expression with the additional 5′ peptide to produce the 312-aa protein with the alternative start codon no longer induced a biased sex ratio. Similarly, expression of a triple-epitope hemagglutinin (HA) tag on either the 5′ end (Fig. S3B) or the 3′ end (Fig. S3C) also ablated the phenotype. Although the HA tags are small (32 aa) and typically do not interfere with protein function, neither tagged version of Wmk induced a biased sex ratio. These results could indicate that the ends are important for function and that inclusion of even small peptides interferes with protein conformation and thus with function, which in turn argues for caution in using epitope-tagged proteins in the study of reproductive parasitism. This supports the result indicating that the alternative start codon does not recapitulate the phenotype either. Although it is not clear why longer forms of Wmk lose function, predicted RNA secondary structures of *wmk* and the 5′ *wmk* gene show that there are significant predicted structural differences with only the additional 27 nucleotides (nt) at the 5′ end and that these differences may affect translation (Fig. S3D).

## DISCUSSION

Previous studies identified genes and gene candidates for reproductive parasitism in *Wolbachia* and *Spiroplasma* bacterial endosymbionts (21, 24–26); however, continued investigations are necessary to fully resolve the genetics of reproductive parasitism for both basic and applied purposes, especially since the diversity of affected hosts is considerable (10, 47). In particular, the evolution of male killing by various microorganisms is hypothesized to be the result of convergent evolution of distinct genes due to differences in the timing of lethality (i.e., early versus late male killing) and in sex determination systems across affected hosts (XY and ZW, for example) (47). Further, *wmk* does not fully recapitulate the phenotype when expressed transgenically. Regarding CI, it also affects a wide variety of hosts, and several diverse phylogenetic types (e.g., types I to IV) of CI genes have been identified (24, 29), leaving open the possibility of the existence of additional types. Here, we evaluated various gene candidates for CI and male killing that were identified in recent studies on reproductive parasitism genetics (Fig. 1). The results and analyses substantiate identification of *cifA*, *cifB*, and *wmk* as the crucial reproductive parasitism genes or gene candidates in phage WO that have been discovered thus far, and the transgenic strains developed here provide a resource to evaluate alternative functions of the tested phage WO genes. Indeed, phage WO genes in the eukaryotic association module are enriched for predicted eukaryotic function or homology, and several of these genes likely interact with the host (22). There are also many genetic aspects of the endosymbiosis that have not been fully explored genetically. These include phage lysis and interactions with the eukaryotic and bacterial host genomes (48), survival of the *Wolbachia* within host cells, other reproductive parasitism phenotypes such as feminization or parthenogenesis, and altered host fecundity (49). Therefore, despite the lack of evidence for involvement in male killing or CI, there are many other possible phenotypes and functions that these transgenic strains could be used to study.

The WD0633 gene was previously investigated in relation to reproductive parasitism (37, 41, 50). The gene is associated with CI genomes (41), contains predicted ankyrin repeats that may mediate interactions with the eukaryotic host (37), encodes a putative type IV effector (see Fig. S2 in the supplemental material) predicted to be architecturally similar to the *Sp*AID male-killing toxin (Fig. 2A; see also Fig. S1B), occurs in a mobile element like the other parasitism genes (21, 24, 25), and is directly adjacent to *cifB* in the EAM (22). However, it is crucially not present in the assembled genome of *w*Rec (23) that causes both CI in its native host Drosophila recens and male killing in the sister species Drosophila subquinaria (11). Despite transgenic assays revealing no role of WD0633 in CI or male killing (Fig. 2), it is possible that, like *cifA* and *cifB*, WD0633 cannot function on its own and may require coexpression with another gene, which will be the subject of future investigations. A homolog exists in many *Wolbachia* genomes, including that of the divergent *w*Bif male-killer strain of Drosophila bifasciata, but it is missing from the reduced genome of *w*Rec (23, 25). The maintenance of the gene in divergent strains and loss in a strain with an eroded phage may suggest that the gene has an important function in phage WO biology, particularly given the likelihood of secretion and interaction with the host. However, any putative functions or strict associations of WD0633 with active phage WO remain to be assessed. In addition, the similarity between the predicted protein structures of *Sp*AID and WD0633 (21) remains a mystery (Fig. S1B).

Although WD0633 and *Sp*AID have no full homologs outside *Wolbachia* and *Spiroplasma*, respectively, the OTU domain within *Sp*AID shares weak homology with domains in three *Wolbachia* proteins other than WD0633. Reciprocally, the domain in WD0633 does not have any non-*Wolbachia* homologs. Thus, it remains possible that there was an old gene transfer event of the OTU domain. The *Sp*AID gene, however, has no known evolutionary connection to sequences within *cifA*, *cifB*, and *wmk*, and there are distinctions between the resulting male killing phenotypes of *Spiroplasma* and *Wolbachia* at the molecular level. For example, the presence of *Spiroplasma* results in neural development defects in D. melanogaster, while *Wolbachia* shows no such defects in D. bifasciata (51). Given these differences and the apparently unique parasitism genes and candidates, it is unlikely that a putative gene transfer from *Spiroplasma* to *Wolbachia* resulted in conferral of a parasitism phenotype.

We also tested the function of *wmk* sequence homologs residing in the *w*Mel genome. Indeed, *w*Mel contains four additional homologs (Fig. 3A), along with two (untested) partial ones that are significantly shortened by transposons and that contain only one HTH domain each (25). Not all reproductive parasitism strains contain additional homologs (*w*Rec has only one), and some have fewer copies than *w*Mel (*w*Inn and *w*Bor have three and *w*Bif has one full and one partial). In addition, *wmk* is the only copy in *w*Mel that has direct homologs in all sequenced male-killing strains based on gene synteny and sequence, making it the best candidate of all copies. In the simplest model, a single *Wolbachia* male-killing gene would be required to kill males. In more-complex models, some or all additional copies may together or individually result in a phenotype. As they are similar homologs and since there are multiple copies in many genomes, we tested the *w*Mel homologs for function. WD0508 (71% sequence homology to *wmk*) was previously demonstrated to not kill males transgenically (25). Here, we showed that the full-length homologs WD0255 (81% sequence homology to *wmk*), WD0622 (65% sequence homology to *wmk*), and WD0623 (81% sequence homology to *wmk*) also did not result in a transgenic phenotype (Fig. 3B). Their functions, if any, therefore remain undetermined, but there are many possibilities. One is that they do not have a function. Since they vary in number from genome to genome, one or a small number may confer function, and the others may represent copies that have lost or not yet gained a function and may not be maintained over time. Another hypothesis is that they may need to work additively. Phenotypes may emerge or strengthen with expression of multiple copies. For example, the moderate penetrance of transgenic *wmk* could be enhanced by one or more of these homologs. Notably, the CI phenotype can be transgenically induced only by dual expression of the *cifA* and *cifB* genes, and neither can induce the phenotype alone (24). However, previous dual expression of *wmk* and the adjacent WD0625 gene did not change the phenotype (25). It is possible that, as a consequence of singly expressing most candidates in this work (including both *wmk* homologs and other candidates), their involvement in a phenotype was missed. However, this is unlikely with the *wmk* homolog candidates since *wmk* is the only full homolog in both the *w*Bif and *w*Rec male-killer genomes that has been shown to induce high levels of male killing (11). Alternatively, additional copies (and perhaps *wmk* as well) may have an unidentified function. Indeed, previous work showed the predicted protein structure of Wmk is similar to that of homologs in other strains despite great amino acid sequence divergence (25). In addition, *wmk* homologs are ubiquitous in *Wolbachia* genomes despite male killing being a rare phenotype. The maintenance of the gene in non-male-killing strains and the conserved predicted protein structure suggest that the Wmk protein may have a pleiotropic function beyond male killing. Indeed, Wmk may have another primary function but can serve as a genetic reservoir for development of male killing in some circumstances. These and additional hypotheses remain to be evaluated in future work.

In addition, it remains unclear why *wmk* has a greater number of divergent copies in some genomes than in others, but this may be important with respect to its ability to adapt to new hosts or to relaxed selection when host resistance suppresses male killing. If so, multiple copies may be correlated with the proclivity of the host for resistance or suppression in cases where male killing may positively affect the fitness of *Wolbachia*. Indeed, *w*Rec and *w*Bif each have only one full-length homolog and *w*Inn and *w*Bor each have three (there is no documented host resistance in *w*Bif, *w*Inn, and *w*Bor), while *w*Mel has five (and may be resistant) (53). Importantly, *w*Bol1b, the male-killing strain of Hypolimnas bolina butterflies, contains seven different homologs. The butterfly host is known to develop resistance in many populations, and this resistance fluctuates in a population over time (17, 52). Thus, the presence of many homologs in this strain also correlates with a known tendency for the evolution of host resistance. In other cases where male killing exists with few homologs (such as *w*Inn), it may be that Wmk targets a protein or peptide that could be lethal to the host if mutated, so resistance may be futile as has been previously hypothesized (53). Further, these three copies are in the same phage WO region with a synteny that matches many other strains, and it is therefore likely that these three copies existed before insertion into these strains by the phage (25). In contrast, *w*Mel and *w*Bol1b have other *wmk* copies not shared by most strains, so they may have uniquely arisen in these prophages. Further, some additional copies in the genomes are the simple result of multiple phage WO insertions with their own version of *wmk* in one *Wolbachia* genome. Fewer copies may therefore correlate with less host resistance, while more copies may correlate with greater resistance, which is the basis of a copy number-host resistance hypothesis. Notably, we can use the negative transgenic expression results associated with these additional copies to narrow down the range of important residues corresponding to the male death phenotype induced by Wmk (the only functional copy thus far). There are 28 residues unique to Wmk that, alone or in combination, may be important for the onset of the phenotype (Fig. 3C). While they are not clustered in any region of the protein, they may help narrow down the points of interactions with putative host targets to be assessed in the future.

Next, we functionally evaluated male-killing candidates identified in our previous comparative genomic analysis (Fig. 4) (25). These candidates were identified by looking for genes shared across the *w*Bif, *w*Inn, *w*Bor, and *w*Rec male-killer genomes, among other criteria. Seven were identified, among which were *wmk* (evaluated previously) and *cifA* (evaluated previously and in the analyses whose results are shown in Fig. 4). Five others remained to be identified (Fig. 4A). Upon transgenic expression, none induced a biased sex ratio (Fig. 4B). They therefore did not recapitulate the phenotype in this system. This does not rule out the possibility of a role in natural parasitism phenotypes conclusively, but it is unlikely they are parasitism genes. It is possible that the transgenic system is not able to fully induce male killing by these genes or that they must be expressed in conjunction with another gene. However, there is no additional evidence (such as homology to toxin-antitoxin systems, etc.) to suggest they function together with *wmk* or any other gene. Of the five genes, WD0627 and WD0628 may be of the most interest for further parasitism research. Their adjacent positions do not necessarily suggest that they function with *wmk* since they are on the opposite DNA strand, which is atypical of cotranscribed genes, although the possibility remains. They are, however, located between *cifA* and *wmk* in the EAM region and are of interest due to their general proximity with these genes.

Given the coassociations of *wmk* and *cifA* in genomics analyses and in physical relation to each other in some divergent genomes (such as *w*Mel and *w*Bif) (24, 25), we hypothesized that the two genes may function together. However, transgenic assays did not demonstrate additive or epistatic effects when they were coexpressed (Fig. 5). Therefore, their relationship with each other, if any, remains unsolved. There are a few possibilities, which are not all mutually exclusive. One is that their colocalization is coincidental due to a putative shared origin. Similarly, they may have close proximity (within ∼5 kb of each other) by chance and not as a consequence of a common origin. Another possibility is that the fitness of *Wolbachia* in a host is contingent upon expression of multiple parasitism phenotypes. If environments are rapidly fluctuating and these phenotypes are subject to pressure as a consequence of environment interactions, multiple genes may enable *Wolbachia* to spread in such circumstances without the cost of relaxed selection with respect to the unexpressed genes. For example, male killing may not be penetrant at high temperatures, but CI might still manifest, allowing the bacteria to proliferate under these conditions (32). Moreover, if one phenotype is ablated due to genetic mutation or host resistance, then the other provides a backup. Two or more genes expressing different phenotypes could function in bet-hedging to benefit *Wolbachia* in complex ecological and environmental scenarios. These or other premises remain to be tested.

As genes beyond *wmk* were evaluated and did not recapitulate CI or male killing, the issue remains of why transgenic *wmk* cannot fully induce a male-killing phenotype and why *w*Mel does not naturally kill males in D. melanogaster. It is still possible that other genes may be involved that have not yet been discovered; however, we have now evaluated all of the top candidates identified thus far. In addition, we previously used many different transgenic drivers to test the premise that different expression levels are required for the phenotype (25). However, increasing the level of expression by an order of magnitude did not result in a change, indicating that expression levels likely do not underlie the partial phenotype (25). The problem represented by the lack of a full transgene phenotype remains unsolved, as does that represented by the lack of male killing via natural infection. One possibility that we explored was that alternative transcripts of *wmk* might underlie a natural lack of phenotype. Inspection of the upstream DNA identified several alternative start codons, in addition to the annotated methionine codon, that are possibly used in embryos (Fig. 6A and B; see also Fig. S3A). Transgenic expression of the most likely alternative transcript yielded no phenotype (Fig. 6C), supporting the hypothesis that alternative transcripts could underlie phenotypic differences.

There is an imperfect correlation with the finding that the male-killer genomes do not have as many alternative start codons as the CI genomes (Fig. 6A). The notable exceptions are the multipotent *w*Rec as well as *w*Bol1b, which infects a butterfly host of ZW sex determination and may not be affected by the same male-killer toxin as drosophilids (XY sex determination) (54). Notably, inspection of the three most commonly used prokaryotic alternative start codons in each of the genomes depicted in Fig. 6A reveals that the *w*Bif and *w*Bor male-killer genomes contain none of these three common codons upstream of the annotated start. Only less-common codons are present. The *w*Inn homolog does contain a GTG codon upstream; in model prokaryotes, however, this is much more commonly used than the annotated ATA codon in this strain (45). Thus, the GTG may be the main start codon, and the ATA codon could represent a misannotation by the Glimmer gene prediction program. All others (the CI strains, the nonparasitic strain, and *w*Bol1b) contain at least one of the top three most commonly used codons upstream of the annotated start. Therefore, of the three natural male-killers in drosophilids, it is possible that none express alternative transcripts or express a very small number. The CI-causing and nonparasitic strains, however, may express higher numbers of longer transcripts that impede function. Indeed, addition of other elements (that do not typically interfere with protein activity) such as an HA tag on either end of the protein was found to ablate the phenotype as well (Fig. S3B and C). This suggests that alternative *wmk* transcripts, which may appear commonly in genomes with certain start codons, may result in loss of function. This therefore represents a hypothesis underlying the natural inability of *w*Mel to kill males in its host, as it may express nonfunctional forms of the protein. Transgenic expression, however, would not encounter this issue as the transcript has only one start codon optimized for host expression. The potential difference in the transcript lengths is one possible reason that transgenic expression of *wmk* results in a phenotype whereas natural expression does not.

The basis of the difference in phenotype from the transcripts is unclear but may lie in potential differences in transcriptional or translational speed affecting the amount of protein. The two phenotypes from the two transcripts could potentially result from different RNA secondary structures impeding protein translation or altering protein folding (see Fig. S3D). Indeed, experiments in Escherichia coli demonstrated that the first 5 to 10 codons in an mRNA transcript greatly determine mRNA folding at the translation start and that this region of mRNA structure is the primary determinant of the translation rate (55). Differing resulting translation rates are proposed to be the basis of selection for noncanonical start codons, as codons would be selected based on their effect on translation. Notably, several of the upstream codons are conserved across several strains, in terms of both codon sequence and location. This conservation in a noncoding region supports the notion of the sequences having putative functional importance, potentially as alternative start codons as tested here or as alternative promoters corresponding to the gene. Future work should compare and contrast the transcripts and putative promoters of various strains to further assess these hypotheses *in vivo*. It remains unclear why *w*Rec would induce male killing in a sister species with the presence of a common alternative codon; however, it is notably unable to kill males in all D. subquinaria strains (11). Therefore, we do not yet understand why the transgenic phenotype is weak, but we present a new hypothesis to account for the difference between the transgenic phenotype and (lack of) native phenotype in which the transgene expresses only the transcript that leads to a sex ratio bias whereas the native strain expresses some number of nonfunctional transcripts.

Here, we evaluated and present evidence on the role of many *w*Mel genes in CI or in male killing or both. The hypotheses according to which those other genes are involved in their tested phenotypes are not supported by the data. Notably, most of the genes were tested singly, and it is possible that they work together to induce a phenotype. Further, *Wolbachia* strains can induce weak versus strong phenotypes (56, 57) or CI versus male killing (11, 12), depending on different factors, including host background. Thus, testing candidate genes in other host genetic backgrounds will be an important future direction that may yield new or different results. Previously, we tested dual *wmk*-WD0625 expression, as those genes had the potential to be coexpressed, but this did not result in a change in phenotype (25). The only two gene candidates within this work that were anticipated to be linked were *wmk* and *cifA* due to the reasons explained above; however, future work may create new dual-expression lines of different gene combinations to determine if additional genes function in parasitism phenotypes. The results also generated several new hypotheses and analyses relating to the connection between *Spiroplasma* male killing and *Wolbachia*, the origin of multiple copies of *wmk* in a genome, Wmk residues critical for protein function, the correlation between *wmk* and *cifA*, and a putative transcriptional basis for some of the complexities of *Wolbachia* genotype and phenotype that could be tested with future work. This work not only advances our understanding of the role of phage WO genes in eukaryotic host biology but will also spur new research into the unique genetics of this symbiosis.

## MATERIALS AND METHODS

### Fly strains and transgene constructs.

The D. melanogaster strains used in this study included several available at the Bloomington *Drosophila* Stock Center, including *Act5c*-Gal4/CyO (BDSC 3953, ubiquitously expressing zygotic driver); y^1^w* (BDSC 1495, *Wolbachia* infected); tetracycline-treated y^1^w* (uninfected), the WT background line of genotype *y*^1^*w*^67c23^; P[CaryP]P2 (BDSC 8622); and *nanos*-Gal4:VP16 (BDSC 4937, gonad-specific driver). In addition, transgene constructs described in our previous publication on *wmk* include WD0034 (control gene) and WD0626 (*wmk*), both of which were codon optimized for *Drosophila* expression and synthesized by GenScript Biotech (Piscataway, NJ) on a pUC57 plasmid, cloned using standard molecular biology techniques into the pTIGER pUASp-based vector for germ line expression that integrates using PhiC31 integrase, and inserted into the BDSC 8622 background line by Best Gene, Inc. (Chino Hills, CA), with transformants selected based on *w^+^* eye color. In addition, previously described constructs included WD0632 (*cifB*, insert line BDSC 8622) and WD0631 [*cifA*, *y^1^ w^67c23^*; P(CaryP)attP40, CytoSite 25C6 insert line from BestGene], which were generated with the same process. The dual WD0631;WD0632 (*cifA;cifB*) line was generated using standard introgression of the two lines. Here, we also describe new transgene constructs. The 3′HA WD0034 (control gene) and 3′HA WD0626 (*wmk*) lines were made with the same process as the constructs described above but were cloned onto the pTIGER-3′HA vector, which includes an additional 3′ triple-HA epitope. WD0622 (BDSC8622), WD0623 (BDSC8622), WD0255 (BDSC8622), WD1243 (BDSC8622), WD0296 (BDSC8622), WD0550 (BDSC8622), WD0633 {BDSC 9736, *y^1^w^1118^*; pBac(y[+]-attP-9A)VK00018 insert line}, WD0627 (BDSC8622), WD0628 (BDSC 9736), 5′HA WD0034 (control gene, triple-HA epitope, BDSC8622), 5′HA WD0626 (*wmk*, triple-HA epitope, BDSC8622), and 5′ WD0626 (*wmk*, 5′ alternative start codon 9 aa upstream, BDSC8622) were all generated via *Drosophila* codon optimization and gene synthesis followed by cloning into the pTIGER plasmid performed by GenScript Biotech (Piscataway, NJ) and subsequent injection and integration of the plasmid into the respective background lines by Best Gene, Inc. (Chino Hills, CA), with transformants selected based on *w^+^* eye color. The dual WD0626;WD0631 (*wmk;cifA*) line was generated using standard introgression of the two lines.

### Fly maintenance.

D. melanogaster flies were reared on a standard cornmeal, molasses, and yeast (CMY) medium. Stocks were maintained at 25°C, with virgin flies stored at room temperature. During collection of virgins, stocks were kept at 18°C overnight and 25°C during the day. All flies were kept on a 12-h light/12-h dark cycle.

### Sex ratio assays.

To assess the effect of transgene expression on adult sex ratios (measurement of male killing), sex ratio assays were performed as previously described (25). Briefly, 20 replicates of 10 uninfected, 4-to-7-day-old virgin female *Act5c*-Gal4/CyO driver flies and 2 uninfected, 1-to-2-day-old virgin male transgene flies were set up in vials with CMY media. They were left on the media to lay eggs for 4 days at 25°C with a 12-h light/12-h dark cycle, at which point the adults were discarded. The vials were then left at 25°C until the offspring were counted. After 9 days of adult offspring emergence, they were scored for both sex and expression (red eye color from *Act5c*-Gal4 chromosome) or nonexpression (curly wings from CyO balancer chromosome). Any vials with fewer than 50 adult offspring were removed from the analysis, as this indicates either poor egg laying or abnormally low egg hatching rates. The number of adult offspring per vial ranged from 50 to 170, with a mean of 120 and a standard deviation of 27.

### Hatch rate assays.

To assess the effect of transgene expression on embryo hatch rates (measurement of CI), hatch rates were determined as previously described (25). Briefly, adult virgin paternal and maternal grandmother females were aged 9 to 11 days before crossing with nonvirgin, non-age-controlled grandfather males of the desired genotype was performed. All uninfected mothers and fathers were derived from crosses between the grandmother nanos-Gal4:VP16 line and either tetracycline-treated y1w* or a transgene grandfather. All infected mothers and fathers were derived from crosses between y1w* (infected) grandmothers and tetracycline-treated y1w* grandfathers. All steps on the maternal side were started 7 days prior to the equivalent step on the paternal side. Mothers were aged 5 to 7 days and fathers were aged 0 to 24 h before the crossings were performed. The fathers in the hatch rate assays are younger than the mothers due to the established CI aging effect, where CI gets weaker as a male ages (58). The mothers and fathers were crossed in single pairs in 8 oz. round-bottom *Drosophila* bottles covered with a grape juice agar plate (created as previously described) with a small smear of yeast paste and tape to hold it down, with 32 to 48 individual crosses per genotype. The bottles were stored with the agar plate down at 25°C overnight (∼16 h), and the grape juice agar plates were swapped for fresh plates supplemented with yeast. The flies were then again stored with the agar plate down at 25°C for 24 h. The plates were then removed, and the parents were discarded. The plates were kept at 25°C except during counting. The embryos on the plate were counted immediately upon removal, and the number hatched was determined again at 36 h. The hatch rate was calculated as the percentage hatched among the total number laid. Any plates with fewer than 25 embryos per mating pair were removed from the analysis, as this indicates poor egg laying. The number of embryos per plate ranged from 25 to 125, with a mean of 50 and a standard deviation of 20.

### Domain and motif analyses.

Protein domains were identified first by running the protein sequences from the NCBI database through SMART (Simple Modular Architecture Research Tool; http://smart.embl-heidelberg.de/) (59) to identify and annotate protein domains. The images produced by this software were used as the basis for the images shown in Fig. 2A and 4A. Additional domains were added if identified in subsequent analysis described here. In addition, the amino acid sequences were run through HHpred (https://toolkit.tuebingen.mpg.de/#/tools/hhpred) (60) to confirm SMART-identified domains and identify additional domain structures in each protein. The analysis was run using default parameters and the following databases: SCOPe70 (v.2.07), COG/KOG (v1.0), Pfam-A (v.32.0), and SMART (v.6.0). Domains were included if they were predicted by SMART and/or represented probabilities greater than 90% in HHpred. The only exception was *Sp*AID, as the ankyrin repeats dominated the results. A second HHpred analysis was done on only the protein sequence after the repeats, which identified the previously reported OTU domain with 94.25% probability.

### T4SS motif identification.

Type IV secretion system (T4SS) effector motifs were identified using S4TE (Searching Algorithm for Type IV Effector proteins, v.2.0; http://sate.cirad.fr/). S4TE is a suite of online bioinformatics tools that analyzes protein sequences for 14 characteristics associated with effectors (such as homology to effectors, eukaryotic domains, subcellular localization signals, etc.) and scores the proteins (39). Those above a threshold value are predicted to be secreted. The analysis was performed by selecting the “*Wolbachia* endosymbiont of Drosophila melanogaster” NCBI genome option and running S4TE 2.0. Of 1,195 total proteins, 148 were above the threshold score of 72. WD0633 returned the highest score of 246 based on its characteristics and is therefore likely to be secreted. The figure generated by the program is presented as Fig. S2 in the supplemental material.

### Alternative start codon identification.

Alternative start codons were identified using Geneious Pro 2019.2.1. The gene sequence of each *wmk* homolog from *w*Mel, *w*Rec, *w*Bif, *w*Inn, *w*Ri, *w*Ha, *w*Bol1-b, *w*Au, and *w*Bor with the additional intergenetic sequence between *wmk* and WD0627 homologs was analyzed. Open reading frames (ORFs) were identified using the Find ORF function in Geneious and the following parameters: a minimum size of 300 nt; including interior ORFs and continued outside sequences; bacterial genetic code; and CTG, ATC, TTG, ATA, ATG, ATT, and GTG as alternative start codons (the default codons of the program). The identified codons are listed in Table S1 in the supplemental material.

### Phylogenetic analyses.

For Fig. S1A, a BLASTP search was done with either the OTU domain of *Sp*AID identified in NCBI (residues 343 to 431) or residues 1 to 342, 432 to 732, and 733 to 1065. Only hits with E values below 1 × 10^−5^ were included in the analysis. All of the hits to the latter three residue regions represented *Spiroplasma* sequences and were not included in further analysis. Results for the OTU domain included three regions from *Wolbachia* sequences and five *Spiroplasma* sequences. These sequences were exported and uploaded into Geneious Pro v.2019.2. The sequences were aligned using MUSCLE (61), and indels were deleted. The alignment was imported to ProtTest v.3.4.2 (62, 63), and the AICc-corrected prediction for best model was cpRev. The MrBayes (64, 65) plugin of Geneious was used to generate a tree with cpRev as the model, and the consensus tree was exported and imported to iTOL v4.4.2 (66), where the final display tree was generated. The same process was used to generate the phylogeny shown in Fig. 3, except that nucleotide sequences were used and JModelTest v.2.1.10 (63, 67) predicted JC to be the AICc-corrected best model; thus, JC was used in the construction of the tree using MrBayes.

### Protein alignment.

The protein alignment presented in Fig. 3C was generated by using a MUSCLE alignment of all sequences in Geneious Pro v.2019.2. Discrepancies in sequences were highlighted, and unique WD0626 (Wmk) sequences were marked manually.

### Statistical analyses.

All statistical analyses of sex ratios and hatch rates were performed using GraphPad Prism 8 software. For sex ratio determinations, a nonparametric Kruskal-Wallis one-way analysis of variance (ANOVA) followed by Dunn’s test of multiple corrections was applied to all gene expression categories, followed by the same test but performed on all nonexpression categories. For hatch rate determinations, a nonparametric Kruskal-Wallis one-way ANOVA followed by Dunn’s test of multiple corrections was applied to all crosses.
