# Supplementary figures and images for: Transgenic Testing Does Not Support a Role for Additional Candidate Genes in Wolbachia Male Killing or Cytoplasmic Incompatibility
Source: mSystems. 2020 Jan 14;5(1):e00658-19. doi: 10.1128/mSystems.00658-19 (PMC6967388; doi:10.1128/mSystems.00658-19)

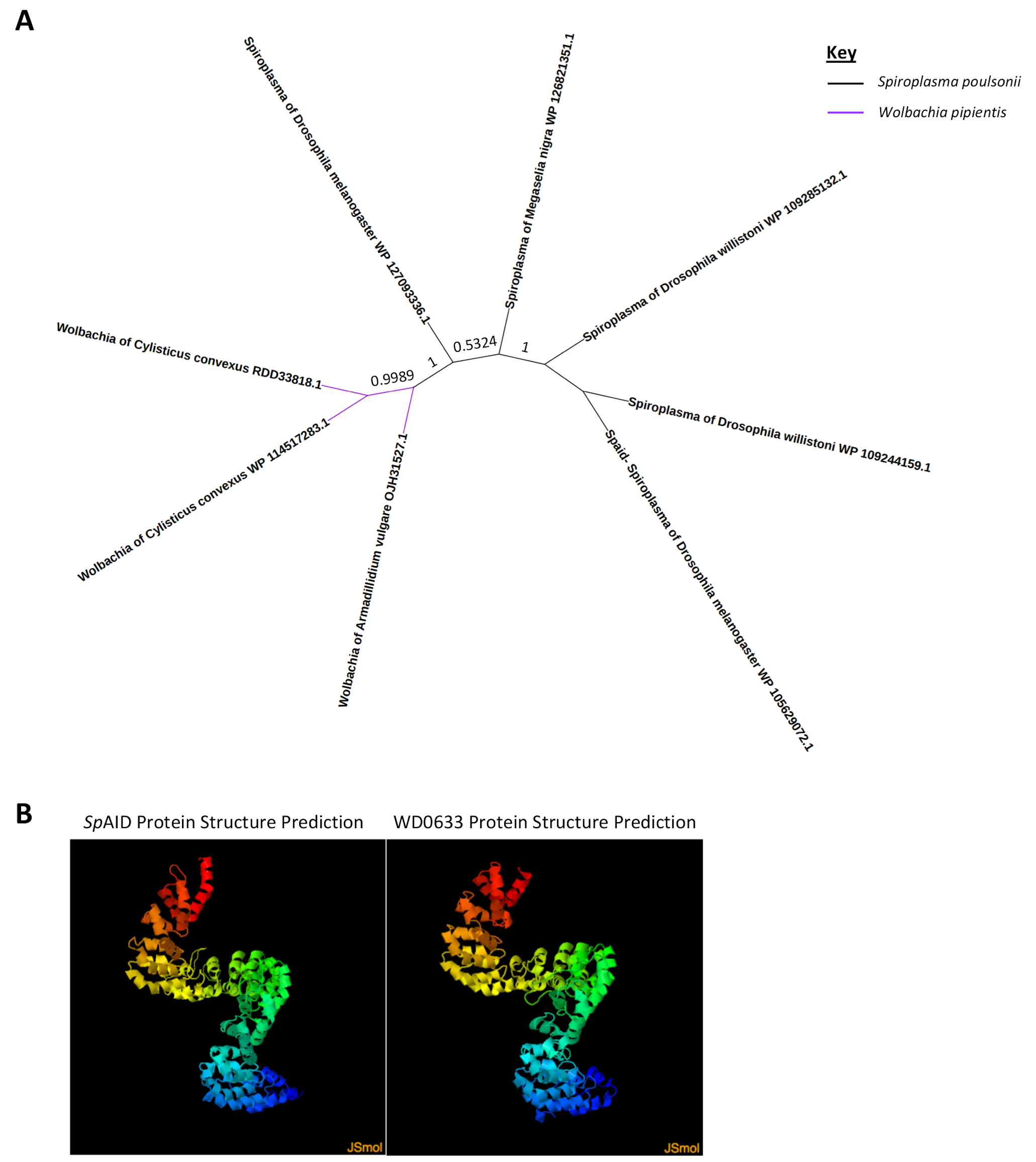

Supplement: FIG S1 [file mSystems.00658-19-sf001.tif]

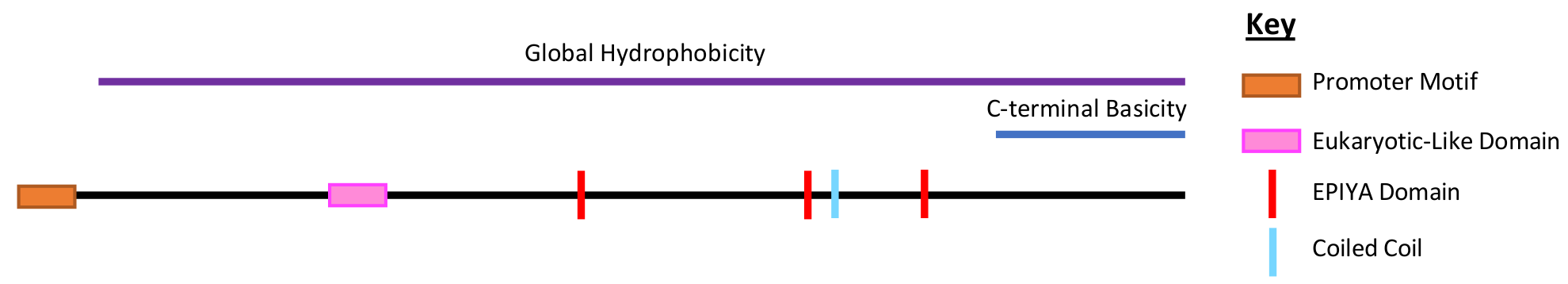

Supplement: FIG S2 [file mSystems.00658-19-sf002.tif]

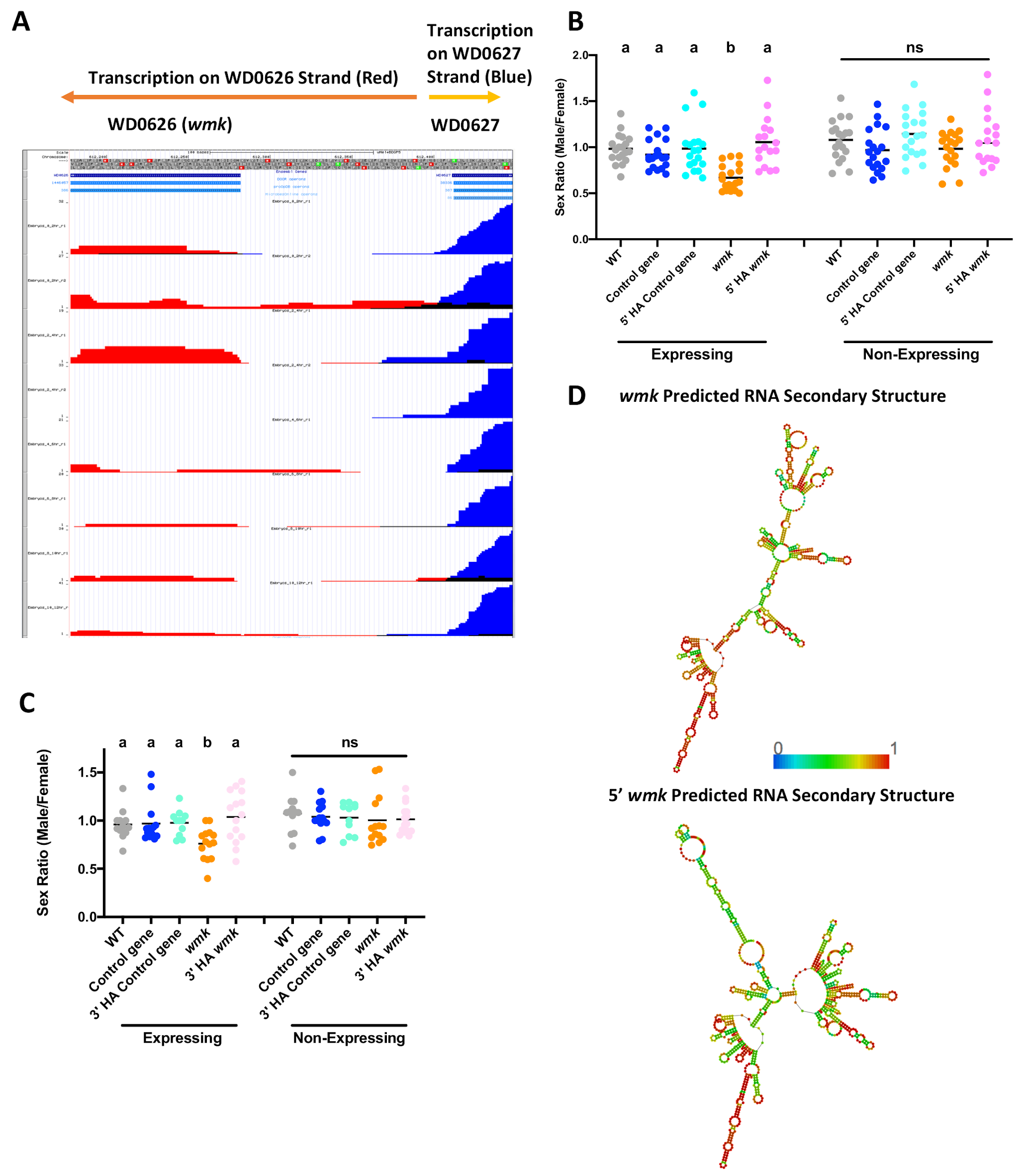

Supplement: FIG S3 [file mSystems.00658-19-sf003.tif]
