# Supplementary material for: Transgenic Testing Does Not Support a Role for Additional Candidate Genes in Wolbachia Male Killing or Cytoplasmic Incompatibility
Source: mSystems. 2020 Jan 14;5(1):e00658-19. doi: 10.1128/mSystems.00658-19 (PMC6967388; doi:10.1128/mSystems.00658-19)
Supplement: TABLE S1 [file mSystems.00658-19-st001.docx]

|  | **Gene Locus Tag (WDXXXX)** | **Score** |
| --- | --- | --- |
| 1 | 0633 | 246 |
| 2 | 0073 | 245 |
| 3 | 0514 | 245 |
| 4 | 0147 | 235 |
| 5 | 0286 | 203 |
| 6 | 0294 | 203 |
| 7 | 0385 | 200 |
| 8 | 0636 | 191 |
| 9 | 0346 | 173 |
| 10 | 0550 | 171 |
| 11 | 0438 | 169 |
| 12 | 0766 | 161 |
| 13 | 0285 | 160 |
| 14 | 1314 | 160 |
| 15 | 0466 | 156 |
| 16 | 0035 | 151 |
| 17 | 0291 | 151 |
| 18 | 0498 | 148 |
| 19 | 1228 | 144 |
| 20 | 0566 | 143 |
| 21 | 0365 | 138 |
| 22 | 0637 | 138 |
| 23 | 0024 | 137 |
| 24 | 0335 | 137 |
| 25 | 0513 | 137 |
| 26 | 0040 | 134 |
| 27 | 0221 | 134 |
| 28 | 0548 | 131 |
| 29 | 0596 | 131 |
| 30 | 0028 | 127 |
| 31 | 0464 | 127 |
| 32 | 0754 | 126 |
| 33 | 0224 | 124 |
| 34 | 0686 | 124 |
| 35 | 0696 | 124 |
| 36 | 0942 | 121 |
| 37 | 1237 | 121 |
| 38 | 0484 | 119 |
| 39 | 1144 | 119 |
| 40 | 0154 | 117 |
| 41 | 0317 | 117 |
| 42 | 0424 | 117 |
| 43 | 0069 | 115 |
| 44 | 0348 | 115 |
| 45 | 0131 | 114 |
| 46 | 0630 | 114 |
| 47 | 0835 | 114 |
| 48 | 1133 | 114 |
| 49 | 1277 | 114 |
| 50 | 0079 | 112 |
| 51 | 0247 | 112 |
| 52 | 0320 | 112 |
| 53 | 0928 | 112 |
| 54 | 1309 | 107 |
| 55 | 0632 | 105 |
| 56 | 0796 | 105 |
| 57 | 1199 | 105 |
| 58 | 0292 | 103 |
| 59 | 0231 | 102 |
| 60 | 0441 | 102 |
| 61 | 0462 | 102 |
| 62 | 0975 | 102 |
| 63 | 1245 | 102 |
| 64 | 0512 | 101 |
| 65 | 0549 | 101 |
| 66 | 1298 | 101 |
| 67 | 0706 | 99 |
| 68 | 0880 | 99 |
| 69 | 1212 | 99 |
| 70 | 0359 | 94 |
| 71 | 0465 | 94 |
| 72 | 0582 | 94 |
| 73 | 0609 | 94 |
| 74 | 0745 | 94 |
| 75 | 0839 | 94 |
| 76 | 0978 | 94 |
| 77 | 1082 | 94 |
| 78 | 1161 | 94 |
| 79 | 1173 | 94 |
| 80 | 0191 | 92 |
| 81 | 0371 | 92 |
| 82 | 0565 | 92 |
| 83 | 0776 | 92 |
| 84 | 1094 | 90 |
| 85 | 0445 | 89 |
| 86 | 0862 | 89 |
| 87 | 0060 | 87 |
| 88 | 0288 | 87 |
| 89 | 0382 | 87 |
| 90 | 0610 | 87 |
| 91 | 0634 | 87 |
| 92 | 0838 | 87 |
| 93 | 1174 | 87 |
| 94 | 0580 | 85 |
| 95 | 1160 | 85 |
| 96 | 0423 | 84 |
| 97 | 0485 | 84 |
| 98 | 0509 | 84 |
| 99 | 0631 | 84 |
| 100 | 0733 | 84 |
| 101 | 1039 | 84 |
| 102 | 1318 | 84 |
| 103 | 0026 | 83 |
| 104 | 0212 | 83 |
| 105 | 0213 | 82 |
| 106 | 0429 | 82 |
| 107 | 0576 | 82 |
| 108 | 0644 | 82 |
| 109 | 0649 | 82 |
| 110 | 0916 | 82 |
| 111 | 1171 | 82 |
| 112 | 1179 | 82 |
| 113 | 1202 | 82 |
| 114 | 1279 | 82 |
| 115 | 0198 | 81 |
| 116 | 0443 | 81 |
| 117 | 0539 | 80 |
| 118 | 1088 | 80 |
| 119 | 0170 | 79 |
| 120 | 0690 | 79 |
| 121 | 0798 | 79 |
| 122 | 0832 | 79 |
| 123 | 1223 | 79 |
| 124 | 0906 | 78 |
| 125 | 0353 | 77 |
| 126 | 0783 | 77 |
| 127 | 0797 | 75 |
| 128 | 0824 | 75 |
| 129 | 1041 | 75 |
| 130 | 1213 | 75 |
| 131 | 0223 | 74 |
| 132 | 1051 | 74 |
| 133 | 1111 | 74 |
| 134 | 0557 | 73 |
| 135 | 0034 | 72 |
| 136 | 0251 | 72 |
| 137 | 0338 | 72 |
| 138 | 0389 | 72 |
| 139 | 0413 | 72 |
| 140 | 0594 | 72 |
| 141 | 0602 | 72 |
| 142 | 0763 | 72 |
| 143 | 0764 | 72 |
| 144 | 0830 | 72 |
| 145 | 0892 | 72 |
| 146 | 0897 | 72 |
| 147 | 0981 | 72 |
| 148 | 1014 | 72 |
