# Supplementary material for: Transgenic Testing Does Not Support a Role for Additional Candidate Genes in Wolbachia Male Killing or Cytoplasmic Incompatibility
Source: mSystems. 2020 Jan 14;5(1):e00658-19. doi: 10.1128/mSystems.00658-19 (PMC6967388; doi:10.1128/mSystems.00658-19)
Supplement: TABLE S2 [file mSystems.00658-19-st002.docx]

| **Genome** | **Codon** | **Additional Length (Amino Acids)** | **Total length (Amino Acids)** |
| --- | --- | --- | --- |
| *w*Mel | ATG* | 0 | 304 |
| *w*Mel | ATA | 2 | 306 |
| *w*Mel | ATA | 3 | 307 |
| *w*Mel | TTG | 9 | 313 |
| *w*Mel | ATC | 16 | 320 |
| *w*Mel | ATA | 20 | 324 |
| *w*Rec | ATG* | 0 | 304 |
| *w*Rec | ATA | 2 | 306 |
| *w*Rec | ATA | 3 | 307 |
| *w*Rec | TTG | 9 | 313 |
| *w*Rec | ATC | 16 | 320 |
| *w*Rec | ATA | 20 | 324 |
| *w*Ri | ATT* | 0 | 304 |
| *w*Ri | ATG | 6 | 310 |
| *w*Ri | ATT | 34 | 338 |
| *w*Ri | ATG | 35 | 339 |
| *w*Ha | ATG* | 0 | 304 |
| *w*Ha | ATA | 2 | 306 |
| *w*Ha | ATA | 3 | 307 |
| *w*Ha | TTG | 9 | 313 |
| *w*Ha | ATC | 16 | 320 |
| *w*Ha | ATA | 20 | 324 |
| *w*Bif | ATG* | 0 | 257 |
| *w*Bif | ATA | 2 | 259 |
| *w*Inn | ATA* | 0 | 313 |
| *w*Inn | GTG | 14 | 327 |
| *w*Bor | GTG* | 0 | 313 |
| *w*Bol1-b | ATG* | 0 | 304 |
| *w*Bol1-b | ATA | 2 | 306 |
| *w*Bol1-b | ATA | 3 | 307 |
| *w*Bol1-b | TTG | 9 | 313 |
| *w*Bol1-b | ATA | 17 | 321 |
| *w*Au | ATG* | 0 | 307 |
| *w*Au | ATA | 2 | 309 |
| *w*Au | ATA | 3 | 310 |
| *w*Au | ATA | 8 | 315 |
| *w*Au | TTG | 9 | 316 |
| *w*Au | ATT | 21 | 328 |
